# Remote ischemic conditioning for acute ischemic stroke part 2: Study protocol for a randomized controlled trial

**DOI:** 10.3389/fneur.2022.946431

**Published:** 2022-08-08

**Authors:** Kentaro Ishizuka, Takao Hoshino, Sono Toi, Takafumi Mizuno, Megumi Hosoya, Moeko Saito, Yasuto Sato, Yoshiki Yagita, Kenichi Todo, Manabu Sakaguchi, Takashi Ohashi, Kenji Maruyama, Shuji Hino, Yutaka Honma, Ryosuke Doijiri, Hiroshi Yamagami, Yasuyuki Iguchi, Teruyuki Hirano, Kazumi Kimura, Takanari Kitazono, Kazuo Kitagawa

**Affiliations:** ^1^Department of Neurology, Tokyo Women's Medical University School of Medicine, Tokyo, Japan; ^2^Department of Public Health, Tokyo Women's Medical University School of Medicine, Tokyo, Japan; ^3^Department of Stroke Medicine, Kawasaki Medical School, Okayama, Japan; ^4^Department of Neurology, Osaka University Graduate School of Medicine, Osaka, Japan; ^5^Department of Neurology, Osaka General Medical Center, Osaka, Japan; ^6^Department of Neurology, Tokyo Women's Medical University Yachiyo Medical Center, Chiba, Japan; ^7^Department of Neurology, Toda Chuo General Hospital, Saitama, Japan; ^8^Department of Neurology, Saitama Red Cross Hospital, Saitama, Japan; ^9^Department of Neurology, Showa General Hospital, Tokyo, Japan; ^10^Department of Neurology, Iwate Prefectural Central Hospital, Iwate, Japan; ^11^Department of Stroke Neurology, National Hospital Organization, Osaka National Hospital, Osaka, Japan; ^12^Department of Neurology, The Jikei University School of Medicine, Tokyo, Japan; ^13^Department of Stroke and Cerebrovascular Medicine, Kyorin University, Tokyo, Japan; ^14^Department of Neurology, Nippon Medical School, Tokyo, Japan; ^15^Department of Medicine and Clinical Science, Graduate School of Medical Sciences, Kyushu University, Fukuoka, Japan

**Keywords:** acute ischemic stroke, remote ischemic conditioning (RIC), neurological severity, good functional outcome, randomized controlled trial

## Abstract

**Background:**

Remote ischemic conditioning (RIC) refers to the application of repeated short periods of ischemia intended to protect remote areas against tissue damage during and after prolonged ischemia.

**Aim:**

We aim to evaluate the efficacy of RIC, determined by the modified Rankin Scale (mRS) score at 90 days after stroke onset.

**Design and methods:**

This study is an investigator-initiated, multicenter, prospective, randomized, open-label, parallel-group clinical trial. The sample size is 400, comprising 200 patients who will receive RIC and 200 controls. The patients will be divided into three groups according to their National Institutes of Health Stroke Scale score at enrollment: 5–9, mild; 10–14, moderate; 15–20, severe. The RIC protocol will be comprised of four cycles, each consisting of 5 min of blood pressure cuff inflation (at 200 mmHg or 50 mmHg above the systolic blood pressure) followed by 5 min of reperfusion, with the cuff placed on the thigh on the unaffected side. The control group will only undergo blood pressure measurements before and after the intervention period. This trial is registered with the UMIN Clinical Trial Registry (https://www.umin.ac.jp/: UMIN000046225).

**Study outcome:**

The primary outcome will be a good functional outcome as determined by the mRS score at 90 days after stroke onset, with a target mRS score of 0–1 in the mild group, 0–2 in the moderate group, and 0–3 in the severe group.

**Discussion:**

This trial may help determine whether RIC should be recommended as a routine clinical strategy for patients with ischemic stroke.

## Introduction

In recent years, hyperacute reperfusion treatment has progressed remarkably due to the establishment of recombinant tissue-type plasminogen activator (rt-PA) and endovascular treatment (EVT) (1, 2). Although Japan is proceeding with a plan to accelerate the development of an efficient care system for stroke patients (forming new stroke centers and stroke care units), only 6–8% of patients can receive hyperacute reperfusion therapy (3). In Japan, edaravone has been used as an effective method to reduce ischemic insults. However, it is not widely used internationally; it has yet to demonstrate sufficient efficacy (4).

Remote ischemic conditioning (RIC) is a therapeutic strategy in which several cycles of brief focal ischemia, followed by reperfusion in the arms or legs, confer protection against the more severe detrimental effects of ischemia in target organs (5–8). Although the underlying mechanisms are not fully understood, current evidence indicates that RIC reduces inflammation, oxidative stress, and cerebral edema, mediated by humoral, immunoregulatory, and neurotrophic factors (9). Although the clinical application of RIC in patients with acute ischemic stroke has been attempted, its efficacy has not yet been validated (10–12). Moreover, no clinical trials have been conducted on acute ischemic strokes in Japan.

One reason for the inability to confirm the efficacy of RIC in previous studies may be the use of a unified definition of a good outcome as assessed by the modified Rankin Scale (mRS), regardless of the neurological severity of the enrolled patients upon admission. Another reason may be the lack of an established RIC protocol. Therefore, this study aims to evaluate the efficacy of an RIC protocol based on recent literature, determined by the mRS score at 90 days after stroke onset, with a good outcome defined according to the severity at enrollment.

## Methods and analysis

### Design

Remote Ischemic Conditioning for Acute Ischemic Stroke (RICAIS) part 2 is an investigator-initiated, multicenter, prospective, randomized, open-label, parallel-group clinical trial targeted at patients with acute ischemic strokes. The protocol is registered with the UMIN Clinical Trial Registry (UMIN000046225).

### Patient population

**Figure 1:**
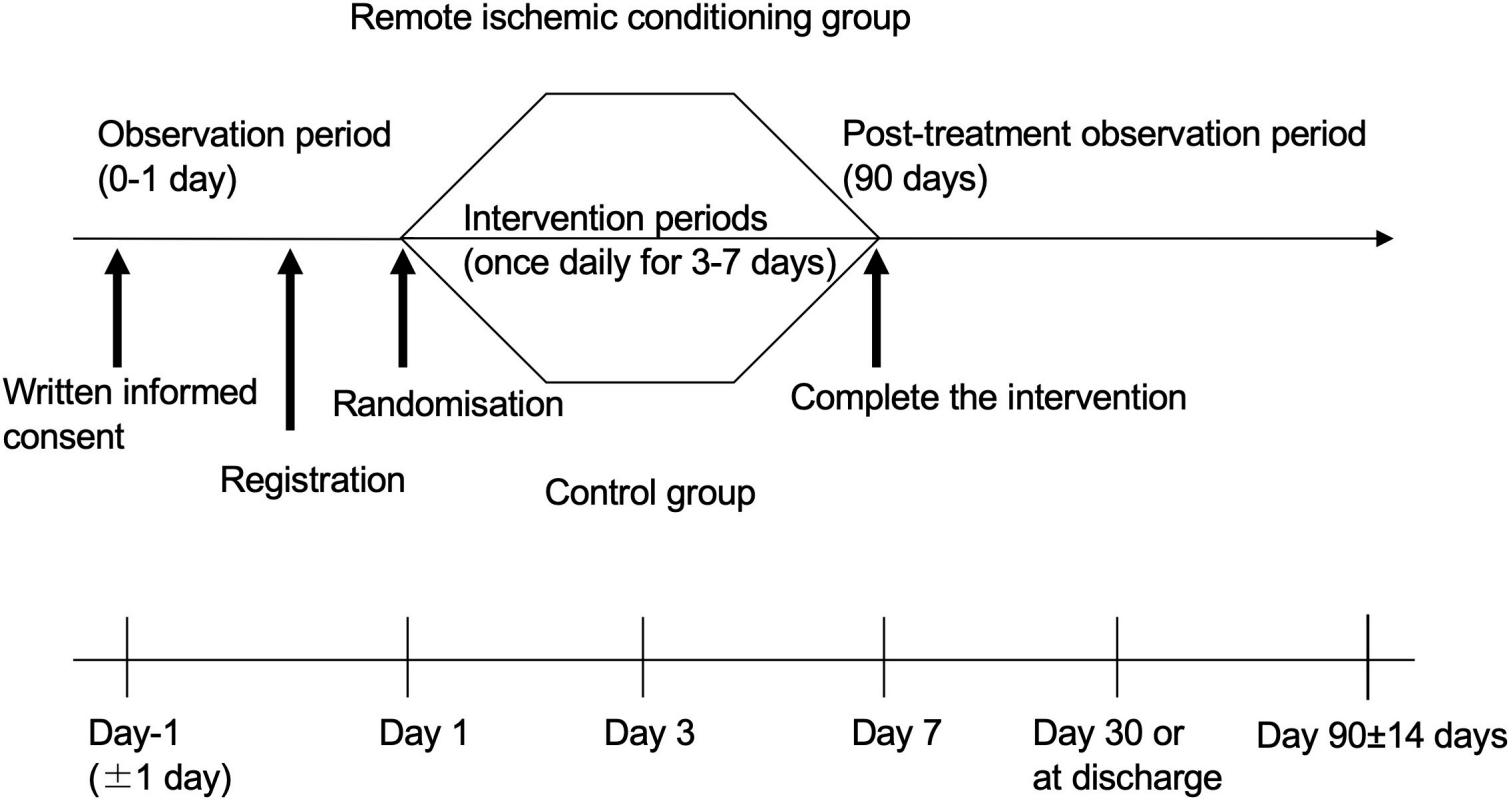
Trial design flowchart.

We will recruit patients diagnosed as having had an acute ischemic stroke *via* brain MRI and/or CT. All 14 Japanese stroke centers and their prehospital regional centers will be invited to participate. The inclusion and exclusion criteria are listed in Table 1, and the baseline assessments and study procedures are provided in Table 2 and Figure 1. Participants need to show a defined ischemic lesion on magnetic resonance imaging (MRI). Patients who received alteplase treatment or mechanical thrombectomy can be enrolled 12 h after the intervention. All patients will provide written informed consent in the acute phase. If the research participant is an adult who is objectively judged to lack the capacity to give informed consent due to a speech or writing impairment caused by a stroke, the consent may be obtained from the legal representative.

**Table 1:** Inclusion and exclusion criteria.

**Inclusion criteria**	**Exclusion criteria**
• Patients hospitalized in participating institutions	• mRS >2 before stroke onset
• Male and female patients (age range, 20–90 years)	• Planned intravenous rt-PA and/or EVT after registration
• Diagnosed as acute ischemic stroke by performing brain MRI and/or CT	• Within 12 h after rt-PA administration or EVT
• Within 48 h after stroke onset	• Systolic blood pressure ≥180 mmHg
• NIHSS scores range from 5 to 20 at registration	• History of PAD
• Tolerance to systemic blood pressure measurement and systolic blood pressure <180 mmHg	• Pregnant patients or patients suspected of being pregnant
	• Patients deemed unsuitable as participants by the investigator

CT, computed tomography; EVT, endovascular treatment; MRI, magnetic resonance imaging; NIHSS, National Institutes of Health Stroke Scale; PAD, peripheral arterial disease; rt-PA, recombinant tissue-type plasminogen activator.

**Table 2:** Study procedures for eligible patients with acute ischemic stroke.

**Procedures/Data collection**	**Screening (days 0–1)**	**Registration/ Baseline visit 1 (day 1)**	**Visit 2 (day 3)**	**Visit 3 (day 7)**	**Visit 4 (day 30 or discharge)**	**Visit 5 (day 90 ±14 days)**
**Enrollment**						
Informed consent	×					
Demographic characteristics	×					
Confirmation of patient background and medical history	×					
Randomization	×					
**Intervention**						
RIC		×	×	×		
**Assessment**						
Concomitant medication confirmation	×	×	×	×	×	×
Physical examination (including height and weight)	×					
Vital signs	×	×	×	×	×	×
Systolic blood pressure measurement of the upper and lower limbs	×	×	×	×		
NIHSS	×	×	×	×	×	
mRS	×				×	×
Questionnaire and pain scale	×	×	×	×	×	×
Description for the case report form	×	×	×	×	×	×
Confirmation of adverse events	×	×	×	×	×	×
Electrocardiogram	×			×		×
**Biomarkers**						
Blood biochemistry	×		×	×		
Brain CT or MRI scan	×			×		

CT, computed tomography; MRI, magnetic resonance imaging; mRS, modified Rankin Scale; NIHSS, National Institutes of Health Stroke Scale; RIC, remote ischemic conditioning.

### Randomization

The patients will be divided according to their National Institutes of Health Stroke Scale (NIHSS) score at enrollment: 5–9, mild; 10–14, moderate; 15–20, severe. Randomization will be performed using a predefined table generated by a computer program, and the results of the assignment will be immediately sent to the physician in charge *via* a computer. To eliminate bias, the assigned study groups will not be disclosed to the evaluating physician during evaluation of mRS at 90 days post stroke. All other staff will have access to the study arm and test results of the participants.

### Intervention

For all patients, a manual blood pressure cuff will be placed around the lower leg or thigh of the unaffected side, and blood pressure will be measured while detecting the dorsalis pedis artery using an ultrasonic Doppler blood flow meter. The intervention (RIC) group will receive four cycles of 5 min of blood pressure cuff inflation, followed by 5 min of reperfusion. Cuff inflation in the RIC group will be set at 200 mmHg or 50 mmHg above the systolic blood pressure; however, if the patient cannot tolerate this, the cuff pressure may be reduced to 180 mmHg. This procedure will be performed once daily after enrollment, for a minimum of 3 days and a maximum of 7 days. In contrast, the control group will only undergo blood pressure measurements before and after the intervention period (the 40-min duration required for four cycles of RIC). Discomfort and pain will be assessed using a verbal rating scale ranging from 0 to 4 (0, no pain; 1, mild pain; 2, moderate pain; 3, severe pain; 4, very severe pain) (13).

### Primary outcome

The primary outcome will be a good functional outcome at 3 months after stroke onset, with a target mRS score of 0–1, 0–2, and 0–3 in the mild, moderate, and severe groups, respectively. The mRS scores will be determined by face-to-face assessments or structured telephone interviews performed by various assessors, who will be blinded to the allocation results.

### Secondary outcomes

The secondary outcomes will be the proportion of patients with good functional outcome (assessed by the mRS) in each group at 3 months after stroke onset; change in NIHSS scores from before RIC to after RIC; incidence of major adverse cardiovascular events (MACE), aspiration pneumonia, and all-cause mortality within 90 days after stroke onset; frequency of adverse RIC-related events; and proportion of patients with an mRS score of 0–1.

### Data monitoring body

Our monitoring staff will be responsible for reviewing the various records of the study and confirming the enrollment status; research plan compliance; completeness, accuracy, and consistency of the data; and compliance with ethical guidelines. The monitoring staff will confirm the contents of the case report forms and ensure research integrity. In addition, the monitoring staff will oversee and manage the condition of each device. The monitoring staff will report any deviations from the protocol, its various procedures, and applicable regulatory requirements to the principal investigator and confirm that appropriate measures are taken and recorded in order to ensure that any identified deviations do not recur. The principal investigator agrees to cooperate with the monitoring staff to ensure that all issues discovered during such monitoring are handled and recorded.

### Sample size calculation

The target sample size is based on previous reports (Table 3). In this study, patients will be divided into three groups based on their neurological severity at enrollment, and the functional outcome at 3 months after stroke onset will be determined for each group. A good functional outcome will be defined as mRS scores of 0–1, 0–2, and 0–3 for the mild (NIHSS score at enrolment, 5–9), moderate (NIHSS score at enrolment, 10–14), and severe (NIHSS score at enrollment, 15–20) groups, respectively. We hypothesize that the rates of good functional outcome are 35 and 50% in the control and RIC groups, respectively; accordingly, with 80% power at the 5% significant level, a sample size of 334 patients is necessary. Furthermore, to account for an expected drop-out rate of 10% (e.g., withdrawal of consent, lost to follow-up), we plan to enroll a total of 400 patients.

**Table 3:** Previous reports on the relationship between the NIHSS on admission and modified Rankin Scale at discharge or 3-months after stroke onset.

**References**	**Year**	**No. patients**	**NIHSS on admission, median (IQR)**	**Severity**	**Therapy**	**mRS (%)**						
						**0**	**1**	**2**	**3**	**0–1**	**0–2**	**0–3**
Thomalla et al. (1)	2018	249	6 (4–9)	Mild	Placebo	15	27	23	17	42	70	87
Lees et al. (14)	2006	847	14.5	Moderate	Placebo	11	20	12	13	31	43	58
Shuaib et al. (15)	2007	1,631	13.8	Moderate	Placebo	10	18	15	15	29	43	58
Shuaib et al. (16)	2011	249	10 (2–20)	Moderate	Control	0	32	12	17	32	44	59
Ginsberg et al. (17)	2013	419	11 (8–17)	Moderate	Placebo	15	23	18	15	38	56	71
Hess et al. (18)	2017	61	13 (8–20)	Moderate	Placebo					12	36	
Saver et al. (19)	2015	843	11.2 (9.8)	Moderate	Placebo	18	16	18	13	35	53	66
Elkins et al. (20)	2019	78	12 (2–29)	Moderate	Placebo	5	15	15	19	20	35	54
Kimura et al. (21)	2003	1,342	0–4							75		94
		1,020	5–9	Mild						41		75
		602	10–14	Moderate						20		46
		521	15–20	Severe						8		23
Goyal et al. (2)	2016	100	<10	Mild	Medical	14	13	28	15	28	55	71
		140	11–15	Moderate	Medical	7	11	16	20	17	34	54
		236	16–20	Severe	Medical	3	4	12	17	8	20	30
Hill et al. (22)	2020	556	17 (13–21)	Severe	Placebo	19	22	19	11	41	59	70
Nogueira et al. (23)	2018	99	17 (14–21)	Severe	Medical	4	5	4	16	9	13	29
Albers et al. (24)	2018	90	16 (12–21)	Severe	Medical	8	4	4	16	12	16	32

IQR, interquartile range; mRS, modified Rankin Scale; NIHSS, National Institutes of Health Stroke Scale; rt-PA, recombinant tissue-type plasminogen activator.

### Statistical analysis

The target populations for analysis will be defined as follows. The full analysis set (FAS) will comprise all randomized participants, with the exclusion of those who never received any protocol treatment, those who underwent at least one protocol treatment but whose post-protocol data are not available, and those who did not meet the eligibility criteria. The intention-to-treat population will comprise all subjects who underwent randomization. The per-protocol set will comprise those in the full analysis set, with the exclusion of subjects with remote ischemia and poor conditioning compliance (<80%). The population for the safety analysis will comprise all subjects who received at least one protocol treatment.

### Statistical analysis of the primary outcome

The primary outcome (a good outcome based on the mRS score at 90 days after stroke onset) will be reported as the percentage. Percentage differences between the RIC and control groups will be evaluated using the Chi-square test. Additionally, multiple regression analysis will be performed. For the multiple regression analysis, corresponding odds ratios and 95% confidence intervals will be calculated, and a *P*-value <0.05 will be considered to be statistically significant.

### Statistical analysis of secondary outcomes

Percentage differences between the RIC and control groups in terms of the incidence of MACE, aspiration pneumonia, frequency of adverse RIC-related events, and all-cause mortality within 90 days will be evaluated using the Chi-square test. The change in NIHSS score from the time of enrollment to 1 week later will be evaluated in a two-sided analysis of variance, with α = 0.05 and power = 0.80. Additionally, with stratified analysis, we will compare the differences in the achievement rate (percentage) of the primary outcome in the RIC and control groups according to the severity of the disease at the time of enrollment (mild, moderate, and severe groups), age (dichotomized by the median of all enrolled patients), sex, presence of occlusion or severe stenosis of the main cerebral artery, treatment with rt-PA and/or EVT, and presence of diabetes mellitus.

## Discussion

Recently, clinical studies of RIC in patients with AIS are attracting a lot of attention. Indeed, several clinical studies are ongoing. The ongoing study by Purroy et al. is a study of acute stroke within 8 h of symptom onset with mRS 0–2 at 90 days as the primary outcome (25). The study of Blauenfeldt et al. is also an ongoing study of acute stroke within 4 h of symptom onset with mRS at 3 months as the primary outcome (26). To our knowledge, our study will be the first clinical trial to investigate the effect of RIC after ischemic stroke in Japan. RIC is safe, feasible, and offers similar clinical benefits to exercise therapy after stroke, while being less physically taxing for the patient (27, 28). This trial may help determine whether RIC should be recommended as a routine clinical strategy for patients with ischemic stroke. RIC may then become one of the choice for therapy in most patients with ischemic stroke. Regarding the pathophysiology for RIC, three potential mechanisms have been proposed: humoral, immunoregulatory, and neurotrophic factors. In addition, several studies indicate that the underlying protective mechanisms of remote ischemic conditioning are associated with its ability to attenuate production of free radicals, promote the cell survival pathway, modulate the immune system, or inhibit the apoptotic cell signaling pathways (29–33). However, these proposed theories still require confirmation.

To date, several clinical studies have evaluated the effectiveness of RIC in patients with acute ischemic stroke, and no study has confirmed its efficacy except for a small clinical trial. Recently, three published clinical studies reported that RIC performed in four cycles of 5 min of inflation and 5 min of deflation for only 1 day after stroke onset did not significantly reduce the brain infarct volume growth or improve the functional outcome at 3 months (10–12). Furthermore, a recent report by Landman et al. studies acute ischemic stroke within 24 h of onset with infarct size on day 4 after admission. In this study, RIC was repeated twice daily with at least 6 h in between and continued for the duration of hospitalization for a maximum of 4 days. However, on average, RIC was conducted for only 2 days and this study did not show significant reduction of the brain infarct size (34). One reason for the inconsistency regarding RIC's effectiveness may be the lack of an established RIC protocol. Previous study by An et al. demonstrated a favorable effect of RIC on the clinical outcome at 3 months using an RIC protocol of five cycles of cuff inflation (to 180 mmHg for 5 min) and deflation (for 3 min) twice per day throughout the duration of the hospitalization (mean, 11.2 days; range, 8–14 days) although this is very small study (35). Additionally, two published experimental studies found that repeated RIC for 14 consecutive days was associated with a smaller infarct size in an animal model for brain ischemia (36, 37). Of these two studies, the study by Ren et al. showed that a single episode of RIC afforded short-term protection including reduction of brain infarct size, while brain infarct size was further ameliorated when combined with repeated RIC during the 14 days after reperfusion (36). Furthermore, a recent animal model study reported that the number and duration of the cycles determined the efficacy of RIC (38). Specifically, two cycles of RIC were insufficient to decrease infarct size, whereas four, six, and eight cycles decreased the infarct size. Similarly, cycles with 2 or 5 min of ischemia decreased the infarct size, whereas cycles with 10 min of ischemia did not reduce the infarct size. In previous experimental studies, three to five 5-min cycles of RIC reduced the infarct size and improved the neurological deficit (33, 39, 40). Our previous experimental study also showed that four cycles of RIC, with each occlusion and release phase lasting 5 min, had efficacy in reducing the infarct volume (41). Based on these reports, it may be essential to perform RIC for a suitable number of cycles and consecutive days to achieve a good functional outcome. Therefore, we will perform four cycles of RIC, alternating 5 min of inflation and 5 min of deflation, repeated over 3–7 days.

The primary outcome of this trial will be good functional outcome at 3 months, determined by the mRS score. In most previous studies, the definition of a good functional outcome at 3 months (based on the mRS) was the same for all patients, regardless of the NIHSS score at stroke onset. Our study differs from previous studies in this respect. Referring to previous studies showing the relationship between NIHSS at admission and mRS at discharge or 3 months after stroke onset, our study will divide patients into three groups based on their neurological severity (assessed by the NIHSS) at enrollment into the trial and will use different target mRS scores to define a good outcome at 3 months according to the severity group. For the safety outcome, the analysis will be based on the entire randomized sample. This approach has been used in a recent comparative study and is considered reasonable given the excellent safety profile of RIC.

Finally, we aim to include 400 patients. In this study, the criteria were simplified to enroll a more significant number of patients with various types of strokes. Therefore, given that ~200 patients with ischemic stroke are admitted to our hospital each year, and since this is a multicenter study, we expect to be able to recruit 400 patients during a recruitment period of 2 years.

## Summary and conclusions

RICAIS part two is a multicenter, prospective, randomized, open-label, parallel-group clinical trial designed to evaluate the efficacy of RIC in patients with acute ischemic stroke. RIC may contribute to improving the functional prognosis by reducing inflammation, oxidative stress, and cerebral edema mediated by humoral, immunoregulatory, and neurotrophic factors. This trial may help determine whether RIC should be recommended as a routine clinical strategy for patients with acute ischemic stroke.

## Ethics statement

The studies involving human participants were reviewed and approved by the Ethics Committee of Tokyo Women's Medical University Hospital (approval number: 2021-0136). The patients/participants provided their written informed consent to participate in this study.

## Author contributions

KKit was the chief investigator and a guarantor. KKit, KI, THo, and ST comprised the steering committees. YS performed the statistical analysis. MSai supervised the data management center. YY, KT, MSak, TO, KM, SH, YH, HY, KKim, THi, YI, TK, RD, TM, and MH were the study investigators. All authors contributed to the article and approved the submitted version.

## Funding

This study was supported in part by the Mihara Cerebrovascular Disorders Research Promotion Fund. The funding source had no role in the design or conduct of the study.

## Conflict of interest

KKit received grants and personal fees from Daiichi Sankyo, Kyowa Hakko Kirin, Bayer Inc., Sanofi, Nippon Boehringer Ingelheim, Takeda Pharmaceutical, and Sumitomo Dainippon Pharma as well as personal fees from Astellas Pharma outside the submitted work. The remaining authors declare that the research was conducted in the absence of any commercial or financial relationships that could be construed as a potential conflict of interest.

## Publisher's note

All claims expressed in this article are solely those of the authors and do not necessarily represent those of their affiliated organizations, or those of the publisher, the editors and the reviewers. Any product that may be evaluated in this article, or claim that may be made by its manufacturer, is not guaranteed or endorsed by the publisher.

## Abbreviations

EVT: endovascular treatment
FAS: full analysis set
MACE: major adverse cardiovascular events
MRI: magnetic resonance imaging
mRS: modified Rankin Scale
NIHSS: National Institutes of Health Stroke Scale
RIC: remote ischemic conditioning
RICAIS: Remote Ischemic Conditioning for Acute Ischemic Stroke
rt-PA: recombinant tissue-type plasminogen activator.
